# *Chlamydia muridarum* Lung Infection in Infants Alters Hematopoietic Cells to Promote Allergic Airway Disease in Mice

**DOI:** 10.1371/journal.pone.0042588

**Published:** 2012-08-01

**Authors:** Malcolm R. Starkey, Richard Y. Kim, Emma L. Beckett, Heidi C. Schilter, Doris Shim, Ama-Tawiah Essilfie, Duc H. Nguyen, Kenneth W. Beagley, Joerg Mattes, Charles R. Mackay, Jay C. Horvat, Philip M. Hansbro

**Affiliations:** 1 Centre for Asthma and Respiratory Disease, School of Biomedical Sciences and Pharmacy, Faculty of Health, University of Newcastle and Hunter Medical Research Institute, Callaghan, Australia; 2 Immunology Program, Garvan Institute of Medical Research, Sydney, Australia; 3 Institute of Health and Biomedical Innovation, Queensland University of Technology, Kelvin Grove, Australia; 4 Experimental and Translational Respiratory Group, School of Medicine and Public Health, Faculty of Health, University of Newcastle, and Hunter Medical Research Institute, Callaghan, Australia; 5 Faculty of Medicine, Nursing and Health Services, Monash University, Clayton, Australia; University Hospital Freiburg, Germany

## Abstract

**Background:**

Viral and bacterial respiratory tract infections in early-life are linked to the development of allergic airway inflammation and asthma. However, the mechanisms involved are not well understood. We have previously shown that neonatal and infant, but not adult, chlamydial lung infections in mice permanently alter inflammatory phenotype and physiology to increase the severity of allergic airway disease by increasing lung interleukin (IL)-13 expression, mucus hyper-secretion and airway hyper-responsiveness. This occurred through different mechanisms with infection at different ages. Neonatal infection suppressed inflammatory responses but enhanced systemic dendritic cell:T-cell IL-13 release and induced permanent alterations in lung structure (i.e., increased the size of alveoli). Infant infection enhanced inflammatory responses but had no effect on lung structure. Here we investigated the role of hematopoietic cells in these processes using bone marrow chimera studies.

**Methodology/Principal Findings:**

Neonatal (<24-hours-old), infant (3-weeks-old) and adult (6-weeks-old) mice were infected with *C. muridarum*. Nine weeks after infection bone marrow was collected and transferred into recipient age-matched irradiated naïve mice. Allergic airway disease was induced (8 weeks after adoptive transfer) by sensitization and challenge with ovalbumin. Reconstitution of irradiated naïve mice with bone marrow from mice infected as neonates resulted in the suppression of the hallmark features of allergic airway disease including mucus hyper-secretion and airway hyper-responsiveness, which was associated with decreased IL-13 levels in the lung. In stark contrast, reconstitution with bone marrow from mice infected as infants increased the severity of allergic airway disease by increasing T helper type-2 cell cytokine release (IL-5 and IL-13), mucus hyper-secretion, airway hyper-responsiveness and IL-13 levels in the lung. Reconstitution with bone marrow from infected adult mice had no effects.

**Conclusions:**

These results suggest that an infant chlamydial lung infection results in long lasting alterations in hematopoietic cells that increases the severity of allergic airway disease in later-life.

## Introduction

Asthma is a chronic inflammatory disease of the airways that is particularly common in children. The clinical course of asthma is punctuated by recurring exacerbations that are underpinned by aberrant CD4+ T helper type 2 lymphocyte (Th2 cell) responses to environmental stimuli [1]. The hallmark symptoms of asthma are mucus hyper-secretion and airway hyper-responsiveness (AHR) that lead to occlusion of the airways, restricted airflow, breathing difficulties and wheezing. These symptoms are driven by the pro-asthmatic Th2 cell-associated cytokines interleukin (IL)-4, -5 and -13 (reviewed in [1], [2]). Viral (particularly rhinovirus and respiratory syncytial virus) and bacterial (particularly *Chlamydia* and *Mycoplasma*) infections in early-life are thought to contribute to the development of asthma [3], [4], [5], [6], [7], [8].

The etiology of asthma suggests that altered immunological programming in early-life by specific infections may play critical roles in the induction and progression of the disease. There is emerging evidence to suggest that both the nature and timing of infection is pivotal in determining whether specific infections protect or predispose to asthma. Numerous studies show an inverse association between Th1-inducing infections and the development of asthma [9], [10], [11]. However, infection with the intracellular bacterium *Chlamydia pneumoniae* is a notable exception and is increasingly linked with the development of asthma in both children and adults [4], [5], [7], [8], [12].

Respiratory infections with *C. pneumoniae* are common and usually asymptomatic but are responsible for up to 22% of all cases of community-acquired pneumonia requiring hospitalization [13], [14]. Significantly, 50–80% of young adults have anti-*C. pneumoniae* antibodies [15], [16], indicating the high prevalence of chlamydial respiratory tract infections in the community during the earlier stages of life. Resolution of infection is mediated by Th1 and interferon (IFN)-γ-driven responses [17], [18]. However, how Th1-inducing chlamydial lung infections are associated with increasing the severity of Th2-mediated asthma remain poorly understood. We have previously shown that *Chlamydia* can infect dendritic cells (DCs) and subvert their function to induce Th2 responses and AHR [19], [20]. We have also shown that the Th2 cytokine IL-13, which is increased in the airways of asthmatics, enhances susceptibility to chlamydial infections in mice [21]. Furthermore we have recently demonstrated that chlamydial lung infection in early-life increases the severity of allergic airway disease (AAD) in later-life [7]. Infection of both neonatal and infant, but not adult, BALB/c mice increased the expression of IL-13 in the lungs, the numbers of mucus secreting cells (MSC) around the airways and AHR during AAD in later-life [7]. We have begun to elucidate the mechanisms involved. Neonatal infection suppressed eosinophilic and Th2-mediated allergic inflammation, but increased systemic DC:T cell IL-13 release and altered lung structure by increasing the size of alveoli [7]. By contrast, infant infection enhanced eosinophilic and Th2-mediated allergic inflammation, but did not alter lung structure [7]. These results suggest that hematopoietic cells may have differential contributions to the mechanisms through which neonatal and infant infections increase the severity of AAD.

Recent studies suggest that hematopoietic cells can respond directly to infections and inflammatory signals [22]. These cells give rise to myeloid and lymphoid immune cell lineages and can proliferate, and differentiate, to replace immune cells lost to cell death following infection. Hematopoietic cells have been shown to sense pathogen components directly via toll-like receptors (TLRs) [23]. Infection-induced, pro-inflammatory cytokine release may also activate hematopoietic cells [24], [25] and aberrant cytokine-induced signalling may have negative effects on the function of these cells [24], [25]. This may have long-term effects on the programming of the immune system and the nature of subsequent responses to antigens.

The effects of chlamydial lung infection on hematopoietic cell function and subsequent AAD have not been investigated. In this study, we demonstrate that reconstitution of bone marrow from mice infected with *C. muridarum* as infants, but not neonates, increases the severity of AAD in later-life. Therefore, early-life infection-induced alterations in hematopoietic cells may play a previously unrecognised role in predisposing to severe AAD.

## Materials and Methods

### Ethics Statement

All experiments were performed with approval from the animal ethics committees of The University of Newcastle and Garvan Institute/St. Vincent's Hospital, NSW.

### Animals

Specific pathogen-free pregnant and non-pregnant BALB/c mice (6, 9, 12 or 15 week old) were obtained from the central animal house, The University of Newcastle or from Australian BioResources (Moss Vale, Australia).

### *C. muridarum* lung infection

Neonatal (<24 hour old), infant (3 weeks old) or adult (6 weeks old) BALB/c mice were infected intranasally with *C. muridarum* (400 [neonate] or 100 [infant and adult] inclusion-forming units, ATCC VR-123, in 5 µl (neonate) or 30 µl (infant and adult) sucrose phosphate glutamate buffer [vehicle]) [6], [7], [26]. Controls were sham inoculated with equivalent volumes of vehicle intranasally.

### Generation of bone marrow chimeras and induction of AAD

**Figure 1:**
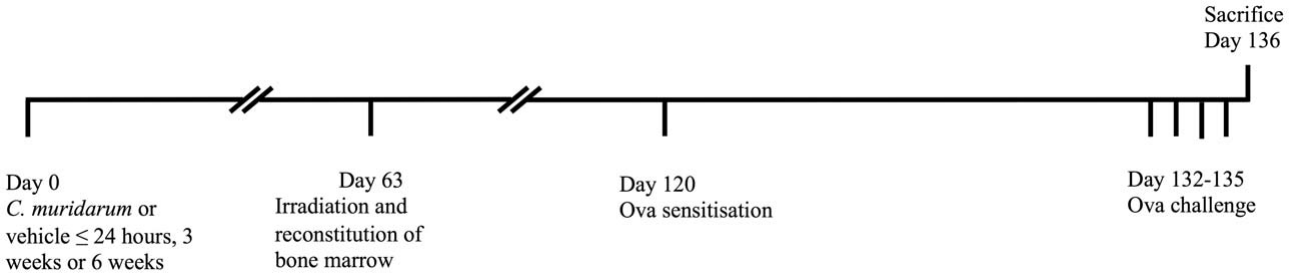
Experimental protocol. BALB/c mice were infected intranasally with *C. muridarum* as neonates (<24 hours old), infants (3 weeks old) or adults (6 weeks old). Controls were inoculated with vehicle (sucrose phosphate glutamate buffer). Nine weeks (63 days) after inoculation, bone marrow was extracted and reconstituted into age-matched irradiated, naive mice. The mice were left for a period of 8 weeks to allow for reconstitution of hematopoietic cells. Mice were then sensitized intraperitoneally (Day 120) to Ovalbumin (Ova) and subsequently challenged 12 days later by intranasal administration of Ova for 4 consecutive days (Day 132–135). Mice were sacrificed 24 hours after the final Ova challenge (Day 136) and features of AAD were assessed.

Nine weeks after neonatal, infant or adult infection, or sham inoculation, bone marrow was extracted from the hind limbs of donor mice and 1×10^7^ cells were intravenously transferred to recipient age-matched irradiated naïve BALB/c mice. Recipient mice were irradiated twice (four hours between each irradiation) with 450RAD (4.5 Gy) prior to adoptive transfer of bone marrow [27]. The mice were left for a period of 8 weeks to allow for reconstitution of bone marrow and hematopoietic cells. It has been shown previously [27], that this method is sufficient for bone marrow reconstitution. AAD was then induced as previously described [6], [7], [28], [29]. Mice were sensitized intraperitoneally to the model allergen Ovalbumin (Ova, 50 µg, Sigma, Missouri, USA) in Rehydrogel (1 mg, Reheis, Berkeley Heights, USA) in sterile saline (200 µl) and subsequently challenged 12 days later by intranasal administration of Ova (10 µg in 50 µl sterile saline) for 4 consecutive days. Mice were sacrificed 24 hours after the final Ova challenge and features of AAD were assessed (Figure 1).

### Determination of mediastinal lymph node cell cytokine release

Mediastinal lymph node (MLN) cells were excised, single cell suspensions prepared, erythrocytes lysed and 1×10^6^ MLN cells plated out in 96 well culture plates in 200 µl of RPMI 1640 culture medium containing 10% FCS, 2 mM L-glutamine, 2 mM sodium pyruvate, 100 µg/ml penicillin, 100 µg/ml streptomycin, 50 µM 2-mercaptoethanol (GIBCO, Invitrogen Mount Waverly, Australia). Cells were re-stimulated with Ova (200 µg/mL; Sigma) for 4 days and the concentration of IL-5, IL-13 and IFN-γ in supernatants were analyzed by ELISA using paired antibodies (R&D systems, Gymea, Australia) as we have previously described [6], [7], [28], [29], [30], [31], [32], [33].

### Quantification of airway MSCs

Lungs were formalin-fixed, embedded, and sectioned (4–6 µm). Sections were stained with periodic acid–schiff for enumeration of MSCs in the airways as we have previously described [32], [34].

### Assessment of lung function

Lung function, in terms of AHR, was measured in anesthetized mice using whole-body plethysmography by determination of transpulmonary resistance and dynamic compliance in response to increasing doses of nebulized methacholine (Sigma) as we have previously described [6].

### Quantification of IL-13 protein in lung homogenates

Whole lungs were homogenized in 1 mL of RIPA buffer (Sigma) and total protein was extracted according to manufacturers recommendations. Extracted total protein from lung homogenates was quantified using BCA Protein Assay Kits (Pierce, Scorsby, Australia). IL-13 protein in lung homogenates was analyzed by ELISA (R&D systems).

### Data analysis

Results are presented as means ± SEM with each experimental group consisting of 6–8 mice, from two independent experiments (3–4 mice per experiment). Each experimental age group was run in parallel with its age-matched control. Between group comparisons of transpulmonary resistance and dynamic compliance (whole curve analysis) were performed using One-way repeated-measures ANOVA. The Mann-Whitney non-parametric test was used for all other comparisons. Analyses were conducted using GraphPad Prism 5 (GraphPad Software, La Jolla, USA). Analysis of statistical outliers was performed using the Grubb's outlier test for GraphPad Prism 5.

## Results

### Establishment of AAD in bone marrow chimeras

**Figure 2:**
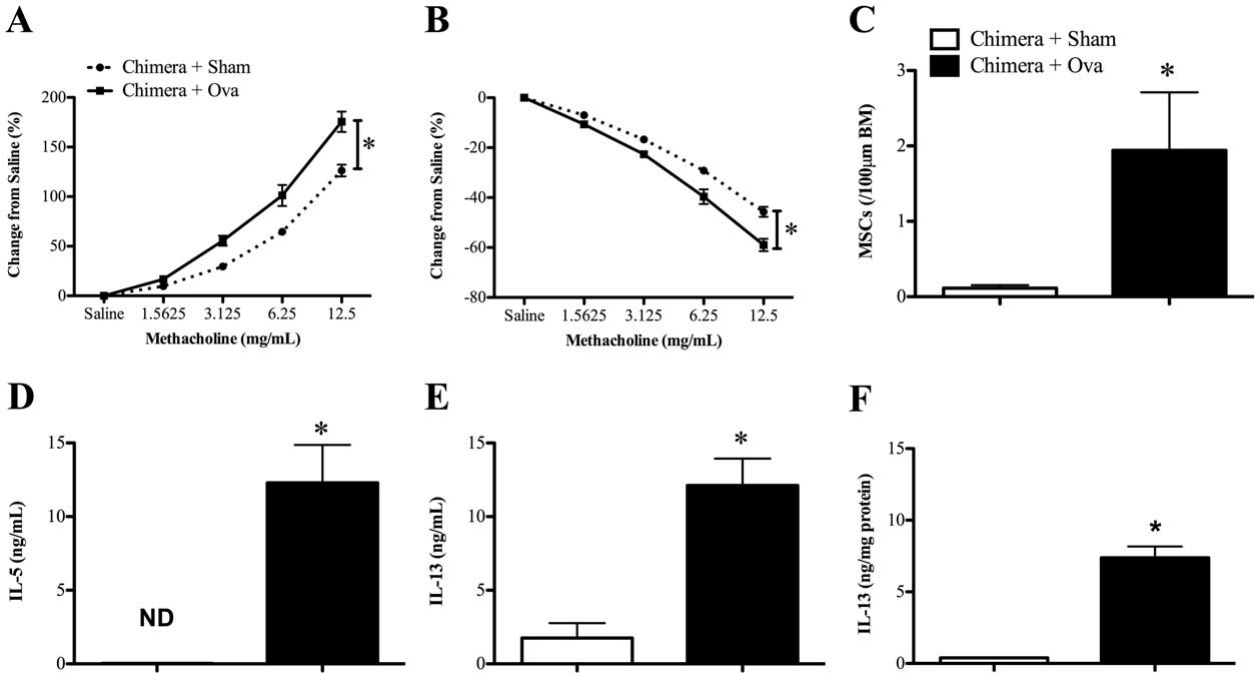
Characterization of AAD in bone marrow chimeras. Bone marrow from adult naïve BALB/c mice was adoptively transferred into age-matched irradiated, naïve mice. Eight weeks later, after bone marrow reconstitution, chimeras were subjected to Ova-induced AAD and the development of AAD was assessed. (**A**) Transpulmonary resistance, (**B**) dynamic compliance, (**C**) mucus secreting cell (MSCs) within 100 µM of basement membrane (BM) in the airways, and Ova-specific (**D**) IL-5 and (**E**) IL-13 release from re-stimulated MLN cell culture supernatants. Results are represented as mean ± SEM. n = 6–8, from two independent experiments of 3–4 mice. *P<0.05 compared to non-allergic (Chimera + Sham) control. ND = not detected.

To establish the chimera system, bone marrow was extracted from naïve (i.e. un-infected) BALB/c mice and transferred to age-matched irradiated naïve mice. After reconstitution of hematopoietic cells (8 weeks later), AAD was induced and assessed. Allergic mice (Chimera+Ova groups) had significantly increased AHR (increased transpulmonary resistance (Figure 2A) and decreased dynamic compliance (Figure 2B)) compared to non-allergic (Chimera+Sham) controls. They also had increased MSC numbers around the airways (Figure 2C) and Ova-specific Th2 (IL-5 and IL-13), but not Th1 (IFN-γ [data not shown]) cytokine release from MLN cells restimulated with Ova (Figure 2D–E). Allergic mice also had significantly increased IL-13 in the lung (Figure 2F) compared to non-allergic (Chimera+Sham) controls. Notably, the development of AAD in chimeras did not involve the influx of eosinophils into the lung (data not shown). These results demonstrate that AAD could be induced after reconstitution of bone marrow.

### *C. muridarum* lung infection in infants, but not neonates, alters hematopoietic cells to increase Ova-specific Th2 cytokine release from MLN cells during AAD

**Figure 3:**
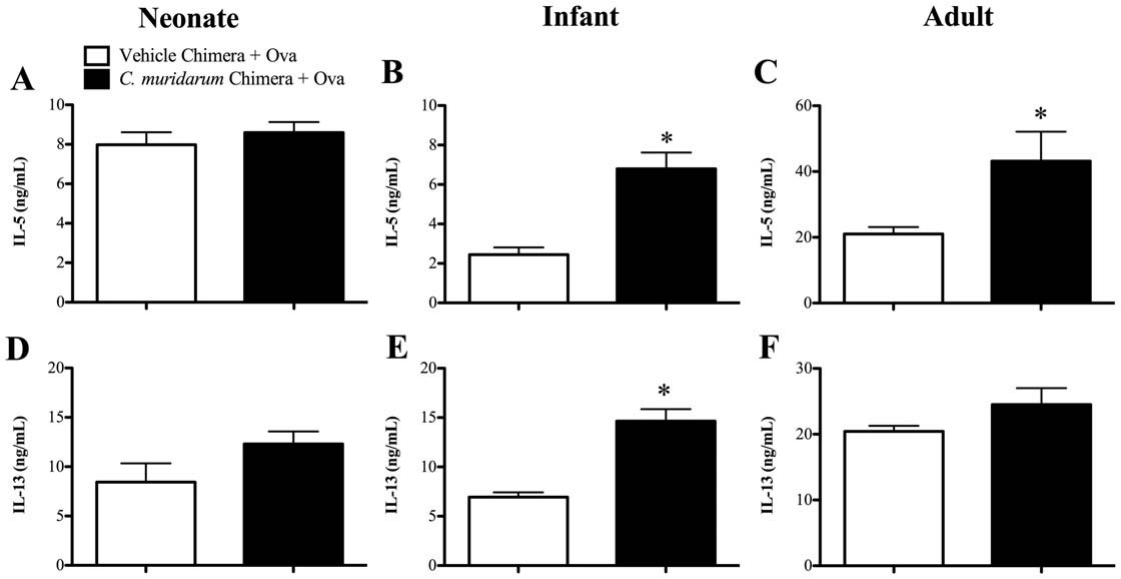
*C. muridarum* lung infection in infants, but not neonates, alters hematopoietic cells to increase Th2 cytokine release from MLN cells. BALB/c mice were infected with *C. muridarum* or sham inoculated (Vehicle) as neonates, infants or adults. Nine weeks after infection bone marrow was extracted and adoptively transferred into age-matched irradiated, naïve mice. Eight weeks after bone marrow reconstitution, chimeras were subjected to Ova-induced AAD and the development of AAD was assessed. Ova-induced (**A–C**) IL-5 and (**D–F**) IL-13 release in MLN cell cultures from neonate, infant and adult bone marrow chimeras. Results are represented as mean ± SEM. n = 6–8, from two independent experiments of 3–4 mice. Age-matched controls were run in parallel with every experiment. *P<0.05 compared to un-infected allergic (Vehicle Chimera + Ova) controls.

We have previously shown that neonatal infection suppressed, whereas infant infection increased Ova-specific Th2 cytokine (IL-5 and IL-13) release from MLN cell cultures, whilst an adult infection had no effect [7]. Here we show that reconstitution of naïve mice with bone marrow from mice infected (*C. muridarum* Chimera+Ova) as neonates had no effect on Ova-specific IL-5 or IL-13 release in AAD compared to age-matched, un-infected allergic (Vehicle Chimera+Ova) controls (Figure 3A). In contrast, reconstitution with bone marrow from mice infected as infants increased IL-5 and IL-13 release from MLN cells in AAD (Figure 3B). Reconstitution with bone marrow from mice infected as adults increased IL-5 but had no effect on IL-13 release (Figure 3C). These results suggest that infant, but not neonatal or adult infection, alters hematopoietic cells within the bone marrow to promote increased Th2 cytokine release in AAD.

### *C. muridarum* lung infection in infants, but not neonates, alters hematopoietic cells to induce MSC hyperplasia during AAD

**Figure 4:**
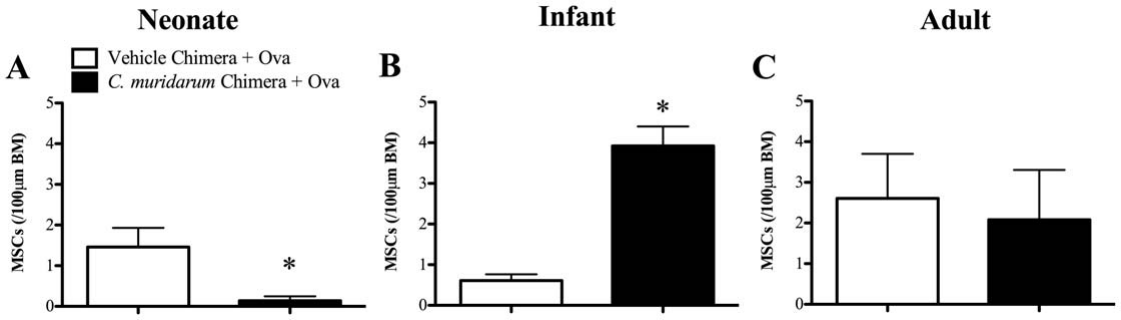
*C. muridarum* lung infection in infants, but not neonates, alters hematopoietic cells to induce MSC hyperplasia. BALB/c mice were infected with *C. muridarum* or sham inoculated (vehicle) as neonates, infants or adults. Nine weeks after infection bone marrow was extracted and adoptively transferred into age-matched irradiated, naïve mice. Eight weeks after bone marrow reconstitution, chimeras were subjected to Ova-induced AAD and the development of AAD was assessed. Numbers of mucus secreting cells (MSCs) within 100 µm of basement membrane (BM) in the airways of (**A**) neonate, (**B**) infant and (**C**) adult bone marrow chimeras. Results are represented as mean ± SEM. n = 6–8, from two independent experiments of 3–4 mice. Age-matched controls were run in parallel with every experiment. *P<0.05 compared to un-infected allergic (Vehicle Chimera + Ova) controls.

We have previously shown that neonatal and infant, but not adult chlamydial lung infection results in increased MSC numbers in the airways during AAD in later-life [7]. Reconstitution of naïve mice with bone marrow from mice infected (*C. muridarum* Chimera+Ova) as neonates resulted in a small but significant reduction, whilst bone marrow from mice infected as infants increased the number of MSCs in the airways in AAD, compared to age-matched, un-infected allergic (Vehicle Chimera+Ova) controls (Figure 4A and 4B, respectively). Reconstitution with bone marrow from mice infected as adults had no effect (Figure 4C). These results suggest that infant, but not neonatal or adult infection, alters hematopoietic cells within the bone marrow, leading to increased MSC numbers in AAD.

### *C. muridarum* lung infection in infants, but not neonates, alters hematopoietic cells to increase AHR during AAD

**Figure 5:**
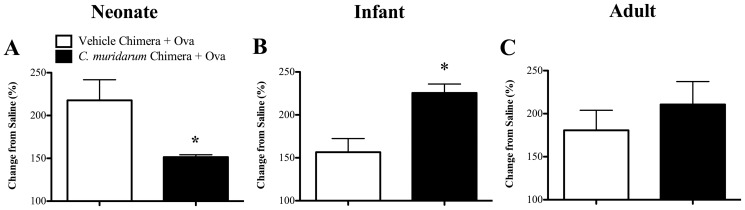
*C. muridarum* lung infection in infants, but not neonates, alters hematopoietic cells to increase AHR during AAD. BALB/c mice were infected with *C. muridarum* or sham inoculated (vehicle) as neonates, infants or adults. Nine weeks after infection bone marrow was extracted and adoptively transferred into age-matched, irradiated naïve mice. Eight weeks after bone marrow reconstitution, chimeras were subjected to Ova-induced AAD and the development of AAD was assessed. Transpulmonary resistance at the maximal dose of methacholine administered (10 mg/mL) in (**A**) neonate, (**B**) infant and (**C**) adult bone marrow chimeras. Results are represented as mean ± SEM. n = 6–8, from two independent experiments of 3–4 mice. Age-matched controls were run in parallel with every experiment. *P<0.05 compared to un-infected allergic (Vehicle Chimera + Ova) controls.

We have previously shown that neonatal and infant, but not adult chlamydial lung infection increases AHR during AAD in later-life [7]. Thus, we investigated whether infection in early-life altered hematopoietic cells to increase AHR. Reconstitution of naïve mice with bone marrow from mice infected (*C. muridarum* Chimera+Ova) as neonates suppressed transpulmonary resistance during AAD, compared to age-matched, un-infected allergic (Vehicle Chimera+Ova) controls (Figure 5A). In stark contrast, reconstitution with bone marrow from mice infected as infants increased transpulmonary resistance (Figure 5B). An adult infection had no effect (Figure 5C). Reconstitution with bone marrow from mice infected as neonates, infants or adults had no effect on dynamic compliance during AAD (data not shown). These results suggest that infant, but not neonatal or adult infection, alters hematopoietic cells within the bone marrow and promotes increased AHR in AAD.

### *C. muridarum* lung infection in infants alters hematopoietic cells to increase IL-13 in the lung during AAD

**Figure 6:**
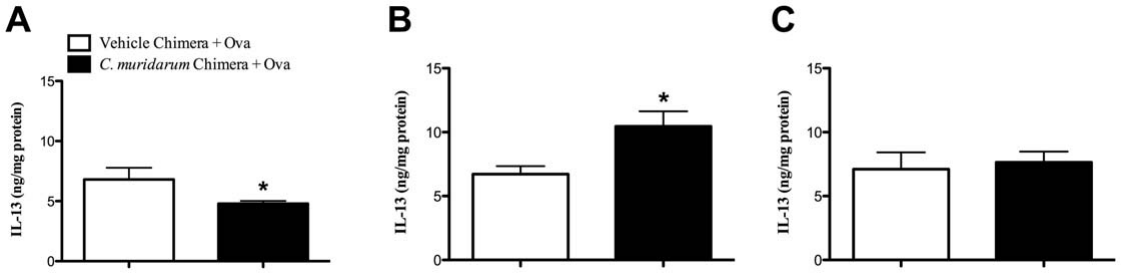
*C. muridarum* lung infection in infants, but not neonates, alters hematopoietic cells to increase IL-13 in the lung during AAD. BALB/c mice were infected with *C. muridarum* or sham inoculated (vehicle) as neonates, infants or adults. Nine weeks after infection bone marrow was extracted and adoptively transferred into age-matched, irradiated naïve mice. Eight weeks after bone marrow reconstitution, chimeras were subjected to Ova-induced AAD and the development of AAD was assessed. IL-13 protein in lung homgenates (**A**) neonate, (**B**) infant and (**C**) adult bone marrow chimeras. Results are represented as mean ± SEM. n = 6–8, from two independent experiments of 3–4 mice. Age-matched controls were run in parallel with every experiment. *P<0.05 compared to un-infected allergic (Vehicle Chimera + Ova) controls.

Given that MLN IL-13 release did not correlate with decreased MSC numbers and transpulmonary resistance in the neonatal group, we assessed IL-13 protein in the lung. Reconstitution of naïve mice with bone marrow from mice infected (*C. muridarum* Chimera+Ova) as neonates suppressed IL-13 production during AAD, compared to age-matched, un-infected allergic (Vehicle Chimera+Ova) controls (Figure 6A). In stark contrast, reconstitution with bone marrow from mice infected as infants increased IL-13 production in the lung (Figure 6B). An adult infection had no effect (Figure 6C). These results suggest that an infant infection alters hematopoietic cells within the bone marrow and promotes increased IL-13 in the lung during AAD.

## Discussion

In this investigation we demonstrate that *C. muridarum* lung infection in infants alters cells in the bone marrow to increase the severity of AAD in later-life. It is most likely that hematopoietic cells are the cells that are affected. We first developed a model of Ova-induced AAD, which has mucus hypersecretion and AHR as hallmark features, in a chimeric system. We then used this model to show that chlamydial lung infection in infants had long lasting effects on bone marrow cells. When bone marrow was transferred into irradiated naïve recipients nine weeks after infant infection, the release of pro-asthmatic Th2-associated cytokines (IL-5 and IL-13) from MLN cells, MSC numbers around the airways, transpulmonary resistance and IL-13 levels in the lung were all increased during Ova-induced AAD. Adoptive transfer of bone marrow from mice infected as neonates had no effect on Th2-cytokine release, and suppressed the number of MSCs around the airways, transpulmonary resistance and IL-13 in the lung. Infection of adult mice had minimal effects. Therefore, an infant, but not neonatal, chlamydial lung infection, increases AAD in later-life through mechanisms that involve alterations in either the number or phenotype of hematopoietic cells. This is the first study to show that the age of infection can have differential effects on hematopoietic cells resulting in either enhanced, or suppressed, AAD in later-life.

In contrast to the affects on transpulmonary resistance, dynamic compliance was unaffected. The large airways are the major contributors to transpulmonary resistance, whereas dynamic compliance is a measure of the elasticity of the small airways and parenchyma [35]. It is likely therefore that infection-induced changes to hematopoietic cells predominantly affected the larger airways in this study. This is supported by the observations that MSC numbers around the large airways were also altered.

There were no differences in all except one of the features of AAD in chimeras generated from sham-inoculated mice. There were no differences between the allergic controls from the three different age groups (Vehicle+Chimera) in terms of MSC numbers, transpulmonary resistance or IL-13 protein in the lung during AAD. This demonstrates that the bone marrow of the chimeras was reconstituted to the same level. The only difference was in the magnitude of MLN cytokine release into culture supernatants. The MLN culture experiments were performed at different times for the different groups. This assay is an *ex vivo* 6 day cell culture assay that is subject to day to day variation and results are compared between groups performed at the same time and not between different experiments carried out at different times. It is not as robust as MSC enumeration, lung function and IL-13 protein in the lung, all of which are direct representations of what occurred *in vivo*.

Notably all mice were adults at the time bone marrow was extracted and reconstituted. Mice infected or sham-inoculated as neonates, infants or adults were 9, 12 and 15 weeks old at time of bone marrow extraction and reconstitution, respectively. Bone marrow was reconstituted into age-matched controls (9, 12 and 15 week old mice for neonate, infant and adult groups, respectively). Each infected group was compared to its relevant age-matched sham-inoculated control, which was run in parallel with each experiment.

For this study we have used an Ova-induced model of AAD, which involves systemic sensitization and airway challenge. Asthma does have a systemic component although it manifests in the respiratory system. We have previously demonstrated that early-life *Chlamydia* lung infection drives more severe Ova-induced AAD in later-life [7]. Thus, the use of this model allowed us to investigate the effects of infection-induced changes in the bone barrow compartment on infection-enhanced AAD in later-life. Our study could be extended by using a house dust mite model of AAD in which sensitization occurs through the respiratory mucosa [34]. Such a study would answer different but important questions that relate to how infection-induced changes in the bone marrow affect mucosal sensitization.

Our results which show that neonatal infection alters hematopoietic cells to suppress, rather than promote the development of AAD in later-life both support and contrast with what we observed in intact (i.e. wild type) mice in our previous studies [6], [7]. In the previous studies neonatal infection also suppressed Th2 and inflammatory responses. However, infection at this age increased MSC and AHR and thus the severity of AAD by inducing systemic IL-13 responses and altering lung structure. The enhanced IL-13 responses involved increased expression in the lung and systemic DC:T-cell release, which correlated with increased MSC numbers and AHR [7]. This contrasts with our current study, which shows that IL-13 protein levels were decreased in the lungs. This indicates that this component of the effects of a neonatal infection does depend on hematopoietic cells. However, we did not investigate the effects of infection on systemic DC:T-cell release in this study. The alterations in lung structure involved alveolar enlargement, which may significantly contribute to changes in lung function [7]. Increased alveolar diameter results in the reduction of alveolar attachments to the airway wall, which decreases airway support and elastic recoil and may lead to enhanced transpulmonary resistance and reduced tissue compliance [36]. The effects on IL-13 responses and lung structure are unlikely to be controlled by the hematopoietic system and in the current study it is not surprising that neonatal infection does not increase the severity of AAD through these processes.

Neonatal infection did not alter the release of IL-5 or IL-13 from Ova-stimulated MLN cells but reduced MSC numbers and AHR, which was associated with attenuated IL-13 production in the lung. It is likely that reductions in IL-13 production in the lung contribute to the reductions in MSC numbers and AHR. Potential explanations for these differential effects in MLNs and lungs are that infection may have differential effects on different compartments so that T-cells in the MLNs can still release sufficient amounts of IL-5 and IL-13, but they may not be able to migrate to the lung to induce MSC hyperplasia and AHR. Another explanation could be that systemic DC and/or T-cell function could be altered. Neonatal infection-induced changes in lung structure in intact wild-type mice is also likely to contribute to infection-induced severe AAD [7].

In contrast to what occurred in neonates, infection of infants altered hematopoietic cells to increase the severity of AAD in later-life by increasing Th2 (IL-5 and IL-13) cytokine release from MLN cells, MSC numbers, transpulmonary resistance and IL-13 production in the lung. This is consistent with what occurs in intact mice, where infection of infants increased the release of the pro-asthmatic Th2 cytokines IL-5 and IL-13, which was associated with increases in MSC numbers and AHR [7]. Interestingly, an infant infection did not result in alveolar enlargement in later-life [7]. Thus, an infant infection may induce long lasting alterations in hematopoietic cells, which drives enhanced AAD in later-life. This study, therefore, extends our previous study by providing evidence of how an infant infection enhances the severity of AAD.

There is mounting evidence that supports a role for infections in altering hematopoietic cells. Hematopoietic progenitors express TLRs that enable these cells to directly sense bacterial components [23]. Upon TLR stimulation these progenitors preferentially differentiate into myeloid cells, which allows for the rapid replenishment of innate immune cells during infection [23]. Hematopoietic cells can also respond to pro-inflammatory cytokines, which may alter their behaviour [22], [37], [38]. Infection may also promote the migration of effector cells back to the bone marrow, which may alter the signature and epigenetic profile of immature bone marrow progenitors. Thus, respiratory *C. muridarum* infection in infants may induce TLR, cytokine and immune cell responses that may promote permanent alterations in the phenotype or activity of hematopoietic cells that drive the development of severe AAD. These possibilities are the subject of ongoing investigations.

In summary, *C. muridarum* lung infection in infancy promotes permanent alterations in hematopoietic cells that drive the development of more severe AAD in later-life. Further studies into the effect of respiratory chlamydial infection on hematopoietic cell differentiation and function, as well as elucidating the role of TLR and cytokine responses, will provide a mechanistic insight into how respiratory chlamydial infections in early-life may increase the severity of AAD in later-life. Such studies may identify new therapeutic targets for the prevention of infection-associated severe asthma.

## References

[1] Hansbro PM, Kaiko GE, Foster PS (2011) Cytokine/anti-cytokine therapy - novel treatments for asthma Br J Pharmacol 163: 81–95. 21232048 10.1111/j.1476-5381.2011.01219.x PMC3085870

[2] Wills-Karp M (2004) Interleukin-13 in asthma pathogenesis. Immunol Rev 202: 175–190. 15546393 10.1111/j.0105-2896.2004.00215.x

[3] Hansbro NG, Horvat JC, Wark PA, Hansbro PM (2008) Understanding the mechanisms of viral induced asthma: new therapeutic directions. Pharmacol Ther 117: 313–353. 18234348 10.1016/j.pharmthera.2007.11.002 PMC7112677

[4] Hansbro PM, Beagley KW, Horvat JC, Gibson PG (2004) Role of atypical bacterial infection of the lung in predisposition/protection of asthma. Pharmacol Ther 101: 193–210. 15030999 10.1016/j.pharmthera.2003.10.007

[5] Hansbro PM, Starkey MR, Kim RY, Foster PS, Horvat JC (2012) Programming of the lung by early life infection. Journal of Developmental Origins of Health and Disease In press. 10.1017/S2040174412000050 25102006

[6] Horvat JC, Beagley KW, Wade MA, Preston JA, Hansbro NG, et al (2007) Neonatal chlamydial infection induces mixed T-cell responses that drive allergic airway disease. Am J Respir Crit Care Med 176: 556–564. 17600276 10.1164/rccm.200607-1005OC

[7] Horvat JC, Starkey MR, Kim RY, Phipps S, Gibson PG, et al (2010) Early-life chlamydial lung infection enhances allergic airways disease through age-dependent differences in immunopathology. J Allergy Clin Immunol 125: 617–625, 625 e611–625 e616. 20122715 10.1016/j.jaci.2009.10.018

[8] Jupelli M, Murthy AK, Chaganty BK, Guentzel MN, Selby DM, et al (2011) Neonatal chlamydial pneumonia induces altered respiratory structure and function lasting into adult life. Lab Invest 91: 1530–1539. 21769086 10.1038/labinvest.2011.103

[9] Thorburn AN, Hansbro PM, Gibson PG (2009) Pneumococcal vaccines for allergic airways diseases. Expert Opin Biol Ther 9: 621–629. 19392578 10.1517/14712590902916999

[10] Thorburn AN, Hansbro PM (2010) Harnessing regulatory T cells to suppress asthma: from potential to therapy. Am J Respir Cell Mol Biol 43: 511–519. 20097830 10.1165/rcmb.2009-0342TR PMC2970851

[11] Kaiko GE, Horvat JC, Beagley KW, Hansbro PM (2008) Immunological decision-making: how does the immune system decide to mount a helper T-cell response Immunology 123: 326–338. 17983439 10.1111/j.1365-2567.2007.02719.x PMC2433332

[12] Patel KK, Vicencio AG, Du Z, Tsirilakis K, Salva PS, et al (2010) Infectious Chlamydia pneumoniae is associated with elevated interleukin-8 and airway neutrophilia in children with refractory asthma. Pediatr Infect Dis J 29: 1093–1098. 21155094 10.1097/inf.0b013e3181eaebdc

[13] Blasi F (2004) Atypical pathogens and respiratory tract infections. Eur Respir J 24: 171–181. 15293621 10.1183/09031936.04.00135703

[14] Vila-Corcoles A, Ochoa-Gondar O, Rodriguez-Blanco T, Raga-Luria X, Gomez-Bertomeu F (2009) Epidemiology of community-acquired pneumonia in older adults: a population-based study. Respir Med 103: 309–316. 18804355 10.1016/j.rmed.2008.08.006

[15] Grayston JT (1992) Infections caused by Chlamydia pneumoniae strain TWAR. Clin Infect Dis 15: 757–761. 1445972 10.1093/clind/15.5.757

[16] Kuo CC, Jackson LA, Campbell LA, Grayston JT (1995) Chlamydia pneumoniae (TWAR). Clin Microbiol Rev 8: 451–461. 8665464 10.1128/cmr.8.4.451 PMC172870

[17] Rottenberg ME, Gigliotti-Rothfuchs A, Wigzell H (2002) The role of IFN-gamma in the outcome of chlamydial infection. Curr Opin Immunol 14: 444–451. 12088678 10.1016/s0952-7915(02)00361-8

[18] Jupelli M, Guentzel MN, Meier PA, Zhong G, Murthy AK, et al (2008) Endogenous IFN-gamma production is induced and required for protective immunity against pulmonary chlamydial infection in neonatal mice. J Immunol 180: 4148–4155. 18322226 10.4049/jimmunol.180.6.4148

[19] Kaiko GE, Phipps S, Hickey DK, Lam CE, Hansbro PM, et al (2008) Chlamydia muridarum infection subverts dendritic cell function to promote Th2 immunity and airways hyperreactivity. J Immunol 180: 2225–2232. 18250429 10.4049/jimmunol.180.4.2225

[20] Beagley KW, Huston WM, Hansbro PM, Timms P (2009) Chlamydial infection of immune cells: altered function and implications for disease. Crit Rev Immunol 29: 275–305. 19673684 10.1615/critrevimmunol.v29.i4.10

[21] Asquith KL, Horvat JC, Kaiko GE, Carey AJ, Beagley KW, et al (2011) Interleukin-13 promotes susceptibility to chlamydial infection of the respiratory and genital tracts. PLoS Pathog 7: e1001339. 21573182 10.1371/journal.ppat.1001339 PMC3088704

[22] King KY, Goodell MA (2011) Inflammatory modulation of HSCs: viewing the HSC as a foundation for the immune response. Nat Rev Immunol 11: 685–692. 21904387 10.1038/nri3062 PMC4154310

[23] Nagai Y, Garrett KP, Ohta S, Bahrun U, Kouro T, et al (2006) Toll-like receptors on hematopoietic progenitor cells stimulate innate immune system replenishment. Immunity 24: 801–812. 16782035 10.1016/j.immuni.2006.04.008 PMC1626529

[24] Belyaev NN, Brown DE, Diaz AI, Rae A, Jarra W, et al (2010) Induction of an IL7-R(+)c-Kit(hi) myelolymphoid progenitor critically dependent on IFN-gamma signaling during acute malaria. Nat Immunol 11: 477–485. 20431620 10.1038/ni.1869

[25] Baldridge MT, King KY, Boles NC, Weksberg DC, Goodell MA (2010) Quiescent haematopoietic stem cells are activated by IFN-gamma in response to chronic infection. Nature 465: 793–797. 20535209 10.1038/nature09135 PMC2935898

[26] Skelding KA, Hickey DK, Horvat JC, Bao S, Roberts KG, et al (2006) Comparison of intranasal and transcutaneous immunization for induction of protective immunity against Chlamydia muridarum respiratory tract infection. Vaccine 24: 355–366. 16153755 10.1016/j.vaccine.2005.07.104

[27] Shum BO, Mackay CR, Gorgun CZ, Frost MJ, Kumar RK, et al (2006) The adipocyte fatty acid-binding protein aP2 is required in allergic airway inflammation. J Clin Invest 116: 2183–2192. 16841093 10.1172/JCI24767 PMC1501108

[28] Preston JA, Essilfie AT, Horvat JC, Wade MA, Beagley KW, et al (2007) Inhibition of allergic airways disease by immunomodulatory therapy with whole killed Streptococcus pneumoniae. Vaccine 25: 8154–8162. 17950502 10.1016/j.vaccine.2007.09.034

[29] Preston JA, Thorburn AN, Starkey MR, Beckett EL, Horvat JC, et al (2011) Streptococcus pneumoniae infection suppresses allergic airways disease by inducing regulatory T-cells. Eur Respir J 37: 53–64. 20525707 10.1183/09031936.00049510

[30] Horvat JC, Starkey MR, Kim RY, Beagley KW, Preston JA, et al (2010) Chlamydial respiratory infection during allergen sensitization drives neutrophilic allergic airways disease. J Immunol 184: 4159–4169. 20228193 10.4049/jimmunol.0902287

[31] Essilfie AT, Simpson JL, Horvat JC, Preston JA, Dunkley ML, et al (2011) Haemophilus influenzae infection drives IL-17-mediated neutrophilic allergic airways disease. PLoS Pathog 7: e1002244. 21998577 10.1371/journal.ppat.1002244 PMC3188527

[32] Essilfie AT, Simpson JL, Dunkley ML, Morgan LC, Oliver BG, et al (2012) Combined Haemophilus influenzae respiratory infection and allergic airways disease drives chronic infection and features of neutrophilic asthma. Thorax 10.1136/thoraxjnl-2011-200160 22387445

[33] Thorburn AN, O'Sullivan BJ, Thomas R, Kumar RK, Foster PS, et al (2010) Pneumococcal conjugate vaccine-induced regulatory T cells suppress the development of allergic airways disease. Thorax 65: 1053–1060. 20965927 10.1136/thx.2009.131508

[34] Thorburn AN, Foster PS, Gibson PG, Hansbro PM (2012) Components of Streptococcus pneumoniae Suppress Allergic Airways Disease and NKT Cells by Inducing Regulatory T Cells. J Immunol 188 In press. 10.4049/jimmunol.1101299 22461699

[35] Vanoirbeek JA, Rinaldi M, De Vooght V, Haenen S, Bobic S, et al (2010) Noninvasive and invasive pulmonary function in mouse models of obstructive and restrictive respiratory diseases. Am J Respir Cell Mol Biol 42: 96–104. 19346316 10.1165/rcmb.2008-0487OC

[36] Saetta M, Finkelstein R, Cosio MG (1994) Morphological and cellular basis for airflow limitation in smokers. Eur Respir J 7: 1505–1515. 7957838 10.1183/09031936.94.07081505

[37] Ueda Y, Yang K, Foster SJ, Kondo M, Kelsoe G (2004) Inflammation controls B lymphopoiesis by regulating chemokine CXCL12 expression. J Exp Med 199: 47–58. 14707114 10.1084/jem.20031104 PMC1887733

[38] Baldridge MT, King KY, Goodell MA (2011) Inflammatory signals regulate hematopoietic stem cells. Trend Immunol 32: 57–65. 10.1016/j.it.2010.12.003 PMC3042730 21233016

